# Predictive Gene Signature of Response to the Anti-TweakR mAb PDL192 in Patient-Derived Breast Cancer Xenografts

**DOI:** 10.1371/journal.pone.0104227

**Published:** 2014-11-06

**Authors:** Ludmilla de Plater, Anne Vincent-Salomon, Frédérique Berger, André Nicolas, Sophie Vacher, Eléonore Gravier, Aurélie Thuleau, Narjesse Karboul, Marion Richardson, Clément Elbaz, Elisabetta Marangoni, Ivan Bièche, Xavier Paoletti, Sergio Roman-Roman, Patricia A. Culp, Bernard Asselain, Véronique Diéras, Didier Decaudin

**Affiliations:** 1 Laboratory of preclinical investigation, Translational Research Department, Institut Curie, Paris, France; 2 Department of Tumor Biology, Institut Curie, Paris, France; 3 Department of Biostatistics, Institut Curie, Paris, France; 4 INSERM U900, Paris, France; 5 Department of Genetics, Institut Curie, Paris, France; 6 Translational Research Department, Institut Curie, Paris, France; 7 AbbVie Biotherapeutics, Redwood City, California, United States of America; 8 Department of Oncogenetic, Institut Curie, Paris, France; Baylor College of Medicine, United States of America

## Abstract

**Purpose:**

(1) To determine TweakR expression in human breast cancers (BC), (2) evaluate the antitumor effect of the anti-TweakR antibody PDL192, used alone or after chemotherapy-induced complete remission (CR), on patient-derived BC xenografts (PDX) and (3) define predictive markers of response.

**Experimental Design:**

TweakR expression was analyzed by IHC on patients and PDXs BC samples. *In vivo* antitumor effect of PDL192 was evaluated on eight TweakR-positive BC PDXs alone or after complete remission induced by a combination of doxorubicin and cyclophosphamide. Using both responding and resistant PDX tumors after PDL192 administration, RT-QPCR were performed on a wide list of selected candidate genes to identify predictive markers of response.

**Results:**

TweakR protein was expressed in about half of human BC samples. *In vivo* PDL192 treatment had significantly anti-tumor activity in 4 of 8 TweakR-positive BC PDXs, but no correlation between the expression level of the Tweak receptor and response to therapy was observed. PDL192 also significantly delayed tumor relapse after CR. Finally, an 8 gene signature was defined from sensitive and resistant PDXs.

**Conclusions:**

PDL192 was highly efficient in some BC PDXs. We found 8 genes that were differentially expressed in responding and resistant tumors and could constitute a gene expression signature which would need to be extended to other xenograft models for confirmation. These data confirm the therapeutic potential of TweakR targeting in BC and the possibility of prospectively selecting patients who might benefit from therapy.

## Introduction

TWEAK (TNFS12, APO3L, or CD255) and its cognate receptor TweakR (Fn14, TNFRSF12A, or CD266), are both members of the Tumor Necrosis Factor (TNF) and TNF receptor (TNFR) super-families, respectively, which are known to be implicated in many important biological processes such as development, hematopoiesis, inflammation, immune response, and tissue repair [1]. Both receptors and ligands of these two families are very attractive targets for therapeutic approaches and many compounds specifically directed against them are either approved or in development for the treatment of cancers or autoimmune diseases [2], [3], [4]. The ligand, discovered in 1997, was named TWEAK (TNF-like weak inducer of apoptosis) because of a weak pro-apoptotic activity on interferon-gamma-treated human HT-29 colon carcinoma cells [5]. Its receptor, a type I transmembrane protein, is able to activate TNFR-associated factors and cytoplasmic proteins that regulate pleiotropic responses, including proliferation, differentiation, and immunoregulatory functions *via* Nuclear Factor-κB (NF-κB) pathway activation [6], [7], [8]. Two types of activation of the TweakR-related signaling pathway have already been described, a first one Tweak-dependent due to the association of the ligand to its receptor, and a second one Tweak-independent due to an overexpression of the receptor. The high expression of the TweakR found in several human cancers when compared with normal tissues [9], [10], [11], [12] may suggest a predominant ligand-independent TweakR signaling, such as in advanced brain tumors [13], [14].

PDL192, a humanized monoclonal antibody directed against TweakR protein, was described to inhibit, *in vitro* and *in vivo,* the growth of various human TweakR-positive cancer cell lines and xenografts. Its antitumor activity is mediated by complex biological pathways, including receptor clustering through Fc effector function and leading to Antibody-Dependent Cell Cytotoxicity (ADCC), NF-kB signaling activation [9], [14]–[17], and proinflammatory cytokines elevation that are associated with human pathology such as pancreatitis [18]–[20]. Hence, it has recently been reported that Tweak Receptor targeting was a promising treatment of breast cancers (BC) [21]–[24]. Here, we provide new data demonstrating the high interest of this new treatment in BC patients.

We first evaluated TweakR protein expression in a large panel of human BC samples and searched for possible clinical correlations and prognostic impact. We secondly evaluated the *in vivo* efficacy of PDL192 in eight well-characterized TweakR-positive patient-derived BC xenografts, alone or after chemotherapy-induced complete remission as maintenance therapy. Finally, using both responding and resistant tumors, we identified 8 genes that were associated with PDL192 response. All of our data showed that TweakR targeting is a promising therapeutic approach in BC patients.

## Materials and Methods

### Ethics Statement

Before PDX establishment, all patients had previously given their verbal informed consent, at time of first consultation at the Institut Curie, for experimental research on residual tumor tissue available after histophatologic and cytogenetic analyses. All patient information was anonymized. Those PDXs establishments have been performed after approval of the ethics committee of the Institut Curie. According to the French rules and the ethics committee of the Institut Curie, a written consent from patients to obtain residual tumor tissues is not required. In case of patient refusal that could be orally expressed or written, residual tumor tissues are not collected. This procedure was approved by ethics committees. This research was not conducted outside of our country. Studies have been performed in compliance with the recommendations of the French Ethical Committee and under the supervision of authorized investigators. The experimental protocol and animal housing were in accordance with institutional guidelines as put forth by the French Ethical Committee (Agreement C75-05-18, France). An ethics committee of the Institut Curie has approved this project and the use of mice. All surgery was performed under xylazin/ketamin anesthesia, and all efforts were made to minimize suffering.

### Human breast cancer samples and patient’s outcome

Tissue-Micro-Array (TMA) banks of human breast tumor samples were established at the Institut Curie with patient consent. These TMAs banks contain 134 breast adenocarcinomas (primaries and/or metastases) allocated in 4 tumor subtypes: basal-like, ERBB2 positive, luminal A, and luminal B breast carcinomas. All clinical data and outcomes of these 134 female patients have been collected and are presented in Table 1. The median age of the population was 52 years (range: 27–89 years) and the median pathological tumor size was 20 mm^3^. All the patients underwent surgery: 99 with tumorectomy (74%), 30 with mammectomy (22%), 4 with pamectomy, and 1 with quadrantectomy. In most cases, surgery was followed by anthracyclin-based chemotherapy (65/134, 48.5%), but 30 patients received an anthracyclin/taxane combination (22%), 3 received taxane alone, and 2.2% other chemotherapies. Radiotherapy was administered to 91% of the population (122/134), 40% received hormone therapy (54/134), and 19% trastuzumab (26/134). With a median follow-up of the overall included population of 46.5 months (range: 2–109 months), the median Disease-free interval (DFS) and overall survival (OS) were 39 and 47 months, respectively. DFS was defined as the time from the diagnosis of breast cancer to the occurrence of a locoregional, distant or controlateral recurrence, metastatic-free survival as the time from the diagnosis to the occurrence of metastasis or death, and overall survival as the time from the diagnosis to the death. Kaplan-Meier survival plots and log-rank tests were used to assess the differences in survival curves [28]. The hazard ratio and its 95% confidence interval (CI) were derived from a Cox proportional-hazards regression model.

**Table 1:** Correlation between TweakR expression and biological and clinical characteristics of the 134 included breast cancer patients.

Characteristics		Overall population				
		N	%	TweakR-positive		P (chi-2; *Wilcoxon)
				N	%	
**Age**	**≤50 years**	54	40	25	46	NS
	**>50 years**	80	60	35	44	
**Pathol. Tumor size (mm)**	**<20**	46	34	23	50	NS
	**20–30**	45	34	15	33	
	**30–50**	25	19	11	44	
	**≥50**	18	13	11	61	
**Invaded lymph nodes**	**0**	55	41	22	40	NS
	**1–3**	46	34	18	40	
	**4–8**	25	19	15	63	
	**>8**	8	6	4	50	
**Initially metastatic tumor**	**Yes**	5	4	2	40	NS
	**No**	129	96	58	45	
**Histological grade**	**I.II**	30	22	14	47	NS
	**III**	104	78	46	44	
**Mitotic index**	**1**	25	19	11	44	NS
	**2**	19	14	10	53	
	**3**	90	67	39	43	
**Vascular emboli**	**Yes**	62	46	31	50	NS
	**No**	72	54	29	40	
**PR expression**	**Yes**	46	35	24	52	NS
	**No**	86	65	36	42	
**ERBB2 expression**	**Yes**	59	44	32	54	NS
	**No**	75	56	28	37	
**Double ER + ERBB2** **expression**	**Yes**	28	21	20	71	**0.012**
	**No**	106	79	40	38	
**Breast cancer sub-groups**	**Basal-like**	39	29	14	36	NS
	**ERBB2-positive**	31	23	12	39	
	**Luminal A**	28	21	12	43	
	**Luminal B**	36	27	22	61	

Pathol. tumor size, pathological tumor size; the mitotic index (MI) was calculated as a percentage as follows: the number of dividing cells divided by the total number of cells present in ten cellular fields (x400).

### Animals and establishment of patient-derived breast cancer xenografts

Breast cancer specimens were obtained with informed consent from the patients at the time of surgery. Fresh tumor fragments were grafted into the interscapular fat pad of 8–12-week-old female Swiss *nude* mice, under xylazin 10 mg.kg^−1^ (Rompun 2%, Bayer-Pharma Santé Animale, Puteau, France)/ketamin 85 mg.kg^−1^ (Panpharma, Fougères, France) anaesthesia. Mice were maintained in specific pathogen-free animal housing (Institut Curie, Paris, France) and received ß-estradiol (8.5 µg.ml^−1^) diluted in drinking water. Xenografts appeared at the graft site about 2 to 8 months after grafting. One xenograft was subsequently transplanted from mouse to mouse and stocked frozen in DMSO-fetal calf serum solution or frozen dried in nitrogen for further studies, and a fragment was fixed in acetic acid, buffered formalin, alcohol solution (Histological Fixer A.F.A. Gurr, VWR, Fontenay-sous-Bois, France) for histological studies [25].

### Immunohistochemistry (IHC)

TweakR immuno-stainings of patients’ tumor TMA and of xenografts were performed using paraffin-embedded sections of tumors fixed in A.F.A. IHC protocol and controls for anti-TweakR staining were provided by Abbott. The melanoma xenografted tumor cell line A375 was used as positive control showing a strong expression of the target, and the negative control was the myeloma xenografted tumor cell line L363 showing no staining. TweakR antibody was used at the 1∶50 dilution after enzymatic treatment and heat. Revelation kit was Leica Menarini (Bond Polymer Refine Detection, n°DS 9800; Visionbiosystem).

Two parameters were used to evaluate TweakR expression: percentage of membranous and/or cytoplasmic epithelial cell staining and staining intensity defined between 0 (no staining) and 3 (high staining). The H-score (histological score) was calculated as *H-score = epithelial cell staining percentage×staining intensity* to integrate these two parameters. Initially, the cut-off of positivity was defined as at least 25% tumor cells with membranous and/or cytoplasmic staining [9]. All stainings were scored by an oncology pathologist.

### Compounds and therapeutic assays

The anti-TweakR monoclonal antibody PDL192 was given thrice a week by intraperitoneous (i.p.) route at a dosage of 10 mg.kg^−1^ after dilution in PBS 1X. The control group was administered with PBS 1X or with a human IgG1 control antibody (MSL109) used as PDL192 isotype control. The combination Doxorubicin, 2 mg.kg^−1^ (Adriamycin, Teva Pharmaceuticals, Paris, France), and cyclophosphamide, 100 mg.kg^−1^ (Endoxan, Baxter, Maurepas, France), diluted in 0.9% NaCl, was given by i.p. route at 3-week intervals. For each drug, toxicity assessments were performed without any loss of weight or diarrhea at chosen dosage. Therapeutic assessments were performed as described previously [25]. Tumor volume was calculated as V = axb^2^/2, a being the largest diameter and b the smallest. Treatment was initiated when tumors in each group achieved an average volume of 60–200 mm^3^. The ratio of each tumor volume at time t to the initial volume was reported as relative tumor volume (RTV). Means (and s.e.) of RTV in the same treatment group were calculated, and growth curves were established as a function of time. Optimal tumor growth inhibition (TGI) of treated tumors vs controls was calculated as the ratio of the mean RTV in treated group to the mean RTV in the control group at each timepoint. Statistical significance of TGI was calculated by the paired Student’s t-test, by comparing the individual RTVs in the treated and control groups. Mice were sacrificed when the tumor volume reached about 2500 mm^3^. Mice were treated and followed up for 120 days.

### Statistical Analysis of TweakR expression in patient BC samples

Association between TweakR expression and clinical characteristics was evaluated by the Pearson’s Chi2 test (or its modifications when conditions for application were not validated). Benjamini and Hochberg corrections [26] were used to adjust for multiple testing. The R software v2.13.2 was used for all statistical analyses [27].

### RNA extraction

Total RNA was extracted from frozen tumor samples by using the acid-phenol guanidinium method. RNA quality was determined by electrophoresis through agarose gels, staining with ethidium bromide and visualization of the 18S and 28S RNA bands under ultraviolet light.

### Real-time RT-PCR

Quantitative values were obtained from the cycle number (Ct value) at which the increase in the fluorescence signal associated with exponential growth of PCR products started to be detected by the laser detector of the ABI Prism 7900 sequence detection system (Perkin-Elmer Applied Biosystems, Foster City, CA), using PE biosystems analysis software according to the manufacturer’s manuals. The primers for genes were chosen with the assistance of the Oligo 6.0 program (National Biosciences, Plymouth, MN). The murine Mm-*TBP* (or the murine target genes) primer pairs and the human Hs-*TBP* (or the human target genes) primer pairs were selected to be unique when compared to the sequence of their respective orthologous gene, whereas the Total-*TBP* primer pair was selected to amplify both the mouse and the human *TBP* genes. dbEST and nr databases were scanned to confirm the total gene specificity of the nucleotide sequences chosen for the primers and the absence of single nucleotide polymorphisms. The nucleotide sequences of the oligonucleotide hybridization primers are shown in Table S3. To avoid amplification of contaminating genomic DNA, one of the two primers was placed at the junction between two exons. Agarose gel electrophoresis was used to verify the specificity of PCR amplicons. The conditions of cDNA synthesis and PCR were as previously described [29]. Transcripts of the *TBP* gene encoding the TATA box-binding protein were quantified as an endogenous RNA control [30] and species specific primers were designed (Table S1).

Results, expressed as N-fold differences in target gene expression relative to the mouse and human *TBP* genes (both the mouse and human *TBP* transcripts) and termed “N*target*”, were determined as Ntarget = 2^ΔCtsample^, where the ΔCt value of the sample was determined by subtracting the average Ct value of target gene (human or mouse) from the average Ct value of ‘Total-*TBP*’ gene). The Ntarget values of the tumor samples were subsequently normalized such that the value for the ‘basal mRNA level’ (Ct = 35) was 1. Target mRNA levels that were absent or very low (Ct>38) in tumor samples were scored “0” (not expressed).

### Proportion of mouse cells in human xenografts

Specific mouse TBP gene expression and the expression of both the mouse and the human *TBP* genes were studied by real-time quantitative RT-PCR using the Mm-TBP as target gene and the Total-TBP as endogenous RNA control. Results, expressed as N-fold differences in specific murine *TBP* gene expression (using Mm-TBP primers) relative to the sum of the mouse and the human *TBP* gene expression (using Total-TBP primers), termed N*Mm-TBP*, are determined by the formula: N*Mm-TBP* = 2^ΔCtsample^. The ΔCt value of the sample is determined by subtracting the Ct value of the murine *TBP* gene from the Ct value of the total (murin + human) *TBP* gene. The N*Mm-TBP* values of the samples are subsequently normalized such that the median of N*Mm-TBP* values of 4 mouse tissues was 100. As *TBP* is a ubiquitously expressed housekeeping gene, showing similar expression in our human and mouse tissues (Ct = 27 for 5 ng cDNA), the final result (normalized N*Mm-TBP* value) determinates the proportion (as a percentage after multiplication by 100) of mouse cell contamination for a given xenograft.

### Quantification of specific mouse (or human) mRNA levels in human xenografts

To quantify by real-time quantitative RT-PCR the specific mouse (or human) mRNA levels in the total RNA, we used the sum of the murine and the human *TBP* transcripts as endogenous RNA control (using the Total-TBP primer pair).

### Determination and application of predictive gene signature

The xenografted models were ranked according to their response to PDL192 in the therapeutic assay described above. After log-transformation and standardization, the expression levels of each gene was compared between the responding and non-responding groups using a two factors analysis of variance (ANOVA) where the factor “model” was nested in the factor “response to treatment” to take into account the intra-model correlation. Cross-validation was implemented: 30 validation subsets were drawn from the original data by sampling without replacement 3 tumor samples per model to maintain the same proportion of each model in the subsets; we repeated the ANOVA on these subsets. We selected genes which were significantly associated with the response to treatment in the training data and 30 validation subsets. Benjamini-Hochberg test was applied to control false discovery rates [26]. The relationships between selected genes were represented by performing an unsupervised classification using hierarchical clustering (Ward Linkage and Spearman correlation coefficient distance used) and the output was visualized by a heatmap. The hierarchical analysis was realized with the R package EMA [31].

## Results

### Tweak-R is widely expressed in human breast cancers, especially in ER-ERBB2 double positive breast carcinomas

**Figure 1:**
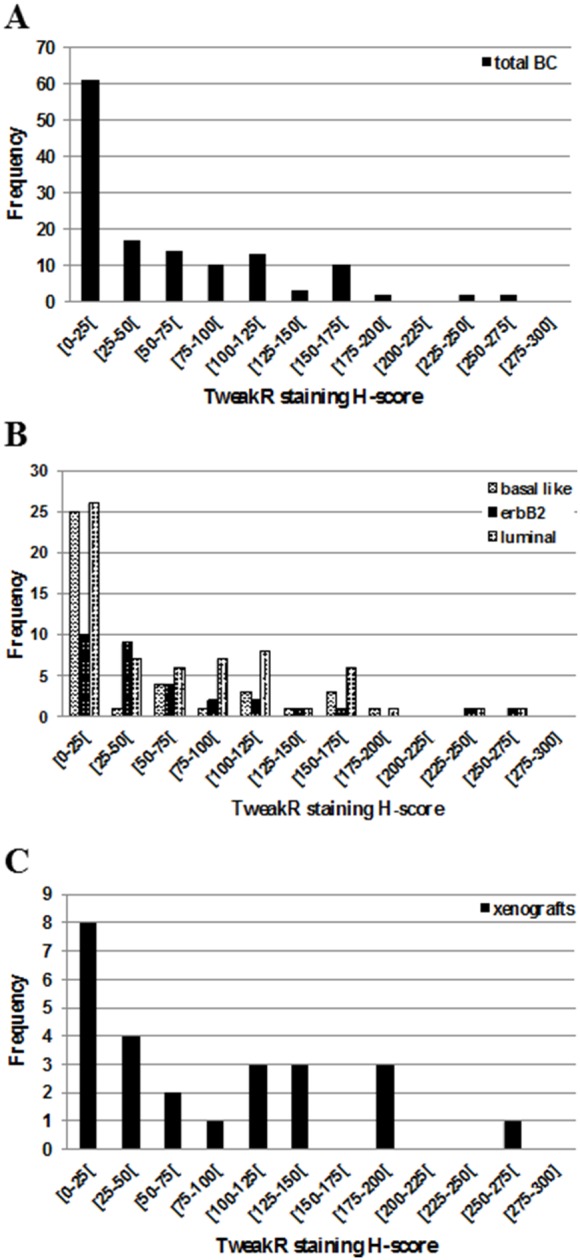
TweakR staining H-score distributions in the overall patient’s tumors (A), in patient’s tumors according to their breast cancer sub-groups (B) and in xenografts (C). H-score was calculated as *H-score = epithelial cell staining percentage×staining intensity*.

**Figure 2:**
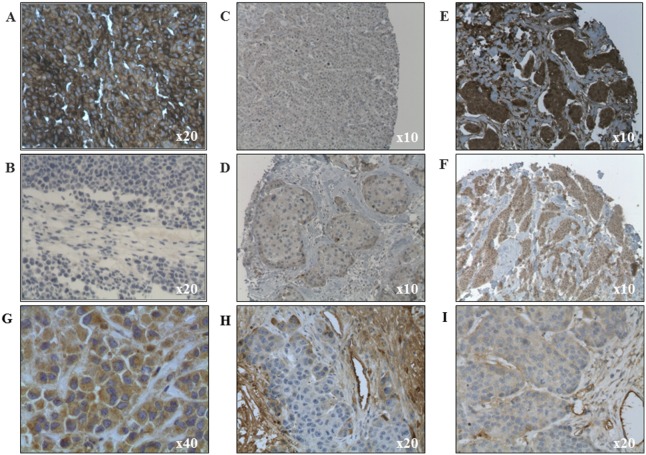
Examples of patient’s tumors and xenografts TweakR expression. **A.** Positive control using the xenografted tumor A375. **B.** Negative control using the xenografted tumor L363 (B). **C to F.** Patient’s tumors showing one negative sample (C), one case of 10%/intensity 3/H-score 30% (D), one case of 30%/intensity 3/H-score 90% (E), and one case of 60%/intensity 2/H-score 120 (F). **G to I.** Xenografts with one case of 95%/intensity 1 to 2/H-score 95% (HBCx-19) (G), one case of 15%/intensity 2/H-score 30% (HBCx-17) (H), and one case of 10%/intensity 2/H-score 20% (HBCx-12B) (I).

Human tumor sample TMAs were screened for TweakR expression by IHC analyses. Using the median H-score of 30 as threshold, TweakR was considered as positive in 12/31 ERBB2 positive carcinomas (39%), 14/39 basal-like tumors (36%), 12/28 luminal A tumors (43%), and 22/36 luminal B tumors (61%), with an overall positive expression in 45% of all tested samples of breast cancer. Most tumor samples presented TweakR cytoplasmic staining without membranous staining (3 membranous staining/134 tumors). The TweakR distribution in term of staining intensity, percentage of positive tumor cells, and H-score is detailed among the overall studied population and among the 3 main breast cancer sub-groups, i.e. luminal, ERBB2, and triple negative tumors (Fig. 1, and Fig. S1). This subgroup distribution was previously determined by clinical investigators. Various examples of TweakR-positive or –negative breast carcinomas are also shown in the Figure 2 (C to F).

In order to define whether TweakR expression is associated with biological or clinical features of breast cancer patients, median H-score threshold was used to interrogate the clinical database corresponding to the human breast cancer TMAs. Numerous clinical features have been considered to analyze their linkage with TweakR expression: age (<50 *v* >50), clinical tumor size (mm) (4 classes: <20, [20]–[30], [30–50], and ≥50), pathological tumor size (mm) (4 classes: <20, [20]–[30], [30–50], and ≥50), pathological number of invaded lymph nodes (4 classes: 0, 1–3, 4–8, and >8), metastatic state M0/M1, Grade I/II *v* III, mitotic index (1-2-3), tumor embolus (yes/no), estrogen receptor (ER), progesterone receptor (PR), ERBB2, molecular subgroups (Basal like (BLC), Luminal A (LA), Luminal B (LB), ERBB2+), EGFR expression (yes/no), Ki67 (<20 *v* >20), P53 protein expression (continuous variable), cytokeratins [8], [18], [5], [6], [14] (continuous variables), and finally the double positive ER+/ERBB2+ group *v* the remaining tumors. As shown in the Table 1, only one of all studied criteria was found to be associated with a high TweakR expression, namely the ER and ERBB2 double positive feature (p = 0.012). No other clinical or biological breast cancer patients’ characteristics were significantly related to TweakR expression. P53 and cytokeratins [8], [18], [5], [6], [14] protein expressions (continuous variables) were also assessed but no correlation to TweakR expression was found (Wilcoxon test, data not shown).

**Figure 3:**
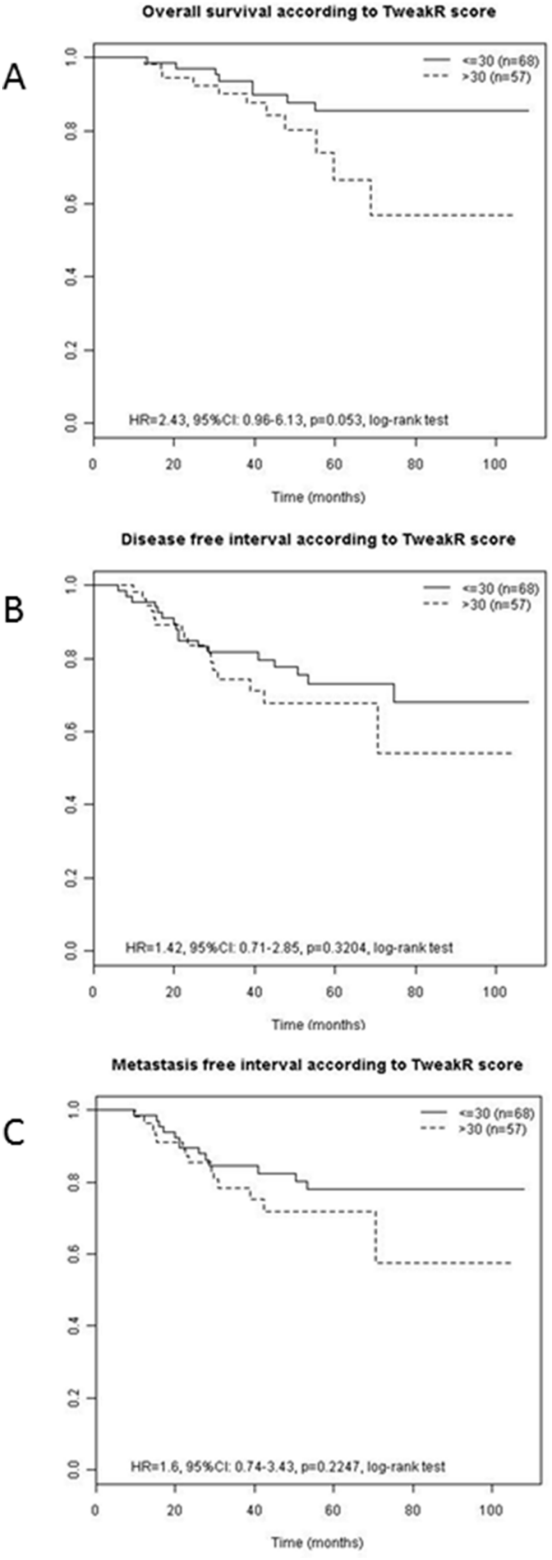
Prognostic impact of TweakR expression on studied BC patients. **A.** Overall survival according to TweakR score. **B.** Disease-free interval according to TweakR score. **C.** Metastasis-free interval according to TweakR score. Kaplan–Meier curves of tumors were determined according to TweakR expression lower (solid line) or higher (dashed line) than median expression of 30. High TweakR expression tends to be significantly associated with a poor overall survival (hazard ratio = 2.43, 95% CI: 0.96–6.13, p = 0.053, log-rank test).

Similarly, in order to define whether TweakR expression possesses a prognostic value by itself, the TweakR H-score was compared to each patient’s outcome. Among the 134 tumors eligible for clinical features, only 125 were eligible for prognosis. The nine removed tumors belonged to women with personal history of cancer whose metastasis were difficult to attribute to their breast cancers, or to women with initial metastatic disease. Prognosis data that were analyzed included overall survival, disease free interval, and metastatic-free interval. Using the median H-score of 30 as threshold, no significant prognostic impact of the TweakR expression was found for disease free survival, or for metastatic-free interval. However, a trend of significance was observed for a negative impact of a high TweakR H-score on the patients’ overall survival (p = 0.053) (Fig. 3), as previously reported [32].

### Tweak-R expression in primary human breast carcinoma xenografts

Twenty-five breast cancer xenograft models were evaluated by IHC for TweakR expression. These PDXs are allocated in 3 tumor subtypes: basal-like (17/25), ERBB2 positive (3/25) and luminal (5/25) breast carcinomas. This imbalance in favor of basal-like subtype is due to the low tumor take of luminal or ErbB2-positive subtypes in mouse [19]. Using the median H-score (i.e. 30) as threshold, TweakR was positive in 16/25 xenografts (64%) (Table S2), 11 xenografts exhibited membranous staining (44%) and 9 cytoplasmic staining (44%). Five xenografts did not show any staining (20%). Moreover, stromal staining was observed in numerous models, mostly on vessels but on fibroblasts in one case. Various examples of TweakR expression in breast carcinomas or PDXs are shown in Figure 2 (G to I). TweakRexpression presented similar distribution between breast cancer TMAs and human breast xenografts in term of percentage of positive tumor cells or H-score, but immunostaining intensity was higher in xenografts with 90% (18/20) of stained xenograft samples presenting an intensity from 2 to 3, whereas only 46% of TMA samples presented a similar intensity (43/93) (Fig. S1 E and F).

In order to define whether TweakR expression was associated with other characteristics of breast cancer xenografts, H-score was compared with various other characteristics of the models, including responses to chemotherapies (doxorubicine, cyclophosphamide, docetaxel, cisplatin, capecitabine) or metastatic potential, but no correlations were found (data not shown).

### PDL192 inhibits tumor growth in multiple human breast carcinoma xenografts

Administered intraperitoneally at a dosage of 10 mg.kg^−1^ thrice a week for 4 weeks, PDL192 was well tolerated and showed no toxicity in treated mice.

**Figure 4:**
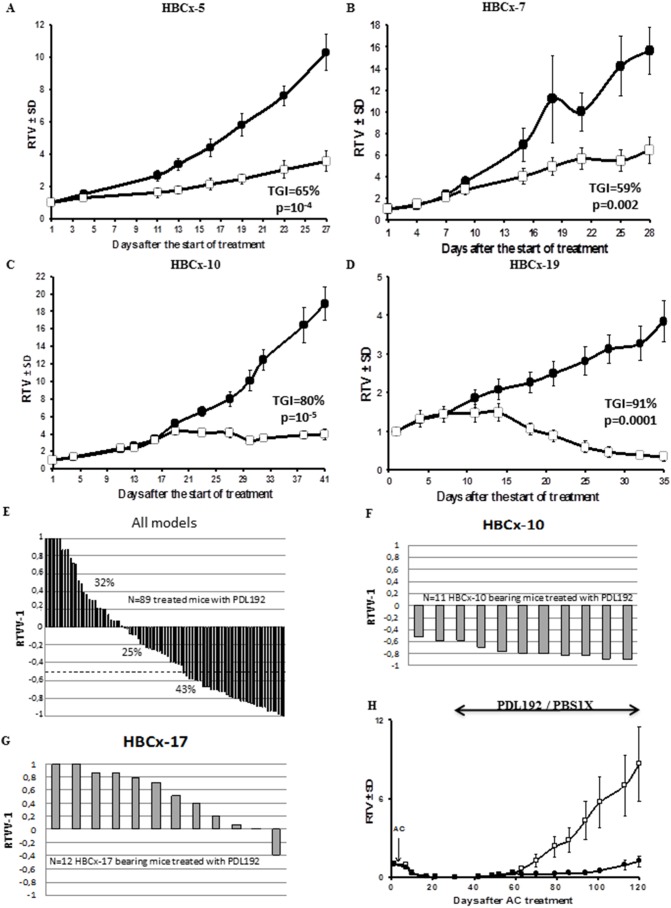
*In vivo* experiments. Mice were treated with PDL192 (□) thrice per week at 10 mg.kg−1. Dosing group contained eight to twelve animals each. Controls (•) were administered by PBS1X. Mice were treated at day 1, and tumor volume was measured twice a week. Tumor growth was evaluated by plotting the mean of the RTV (relative tumor volume) ± SD per group over time after first treatment. **A to D.** Responding xenografts including HBCx-5 (A), HBCx-7 (B), HBCx-10 (C), and HBCx-19 (D). **E to G.** Relative variations of all treated tumors. Growth curves were obtained by plotting mean RTV against time. All PDL-192-treated tumors are included in the analysis (E). Examples of the responding xenograft HBCx-10 (F) and the non-responding xenograft (G). **H.** PDL192 used in an adjuvant setting after chemotherapy-induced complete remission (HBCx-10). After doxorubicin-cyclophosphamide administration, mice were randomized into two groups: one group was treated thrice per week with PDL192 (10 mg.kg^−1^ for 13 weeks) (•), and one control group treated by PBS1X (□).

Nine TweakR-positive breast cancer PDXs with at least 10% of positive-TweakR tumor cells were chosen for PDL192 treatment. As shown in Figure 4A–D, four models among the nine PDL192-treated xenograft models presented significant tumor growth inhibition (TGI) greater than 50% (44%). HBCx-5, -7, -10, and -19 were responding to PDL192 treatment with TGIs of 65% (p = 10^−4^), 59% (p = 0.002), 80% (p = 10^−5^), and 91% (p = 0.0001), respectively. Moreover, the HBCx-19 xenograft showed four out of ten treated mice with complete tumor regression at day 35. The four responding models belonged to two different breast carcinoma subtypes: three were basal like carcinomas (HBCx-7, HBCx-10, and HBCx-19) and one was an ERBB2-overexpressing breast carcinoma (HBCx-5). In these four xenografts, significant differences in tumor volumes (p≤0.05) between control and PDL192 treated groups appeared late, around day 20, but were constant until the end of dosing. No correlation was observed between previously defined characteristics of the xenografts and the *in vivo* efficacy of PDL192, and particularly the TweakR H-score value (Table S3).

To evaluate responses to PDL192 antibody according to individual mouse variability, and show PDL192 efficacy using waterfall plot representation, each mouse was considered to be one tumor-bearing entity. Hence, in all *in vivo* experiments, a relative tumor volume variation (RTVV) of each treated mouse was calculated from the following formula: [(Vt/Vc)–1], where Vt is the volume of the treated mouse and Vc the median volume of the corresponding control group at a time corresponding to the end of treatment. Then, for each treated mouse, we calculated [(RTVV)-1]. A tumor was considered as responding to therapy when [(RTVV)-1] was lower than −0.5. When relative tumor volume variation [(RTVV)-1] of each PDL192-treated mouse was calculated, we observed that 68% of all PDL192-treated mice (60/89) had a negative ratio compared to control groups, and that 43% had a ratio lower than 50%, (Fig. 4E), which constitutes the threshold to consider a model as responding. Within a given model, the response was quite homogeneous, as illustrated by the individual animals’ responses in the HBCx-10 and HBCx-17 models (Fig. 4F and 4G). In the HBCx-10 model, 11/11 mice responded, while in the HBCx-17 model, 0/12 responded.

### Efficacy of PDL192 used as maintenance therapy after chemotherapy-induced complete remission

We next addressed the role of TweakR-positive cells in initiating tumor recurrences after conventional therapy, as previously described [33]. Mice implanted with the basal-like breast cancer model HBCx-10 were treated with doxorubicin-cyclophosphamide to induce complete remissions, after which they were administered PDL192, i.p. at a dosage of 10 mg.kg^−1^ thrice a week for 13 weeks. In this setting, PDL192 significantly delayed tumor relapses (Fig. 4H). At the end of treatment (day 120), 4/10 (40%) PDL192-treated mice presented tumor relapses versus 7/11 (63%) in the control group. Moreover, the median time to relapse was significantly longer in treated mice than in the control group: 66 days (range: 52–113 days) in the control group *versus* 116 days (range: 66–128 days) in the PDL192-treated group (p = 0.046) (Fig. 4H). Interestingly, such a result was observed in a low TweakR-positive model (H-score = 20), suggesting that other host or tumor factors may impact the *in vivo* efficacy of PDL192.

### Determination of differentially expressed genes between responding and resistant tumors

**Figure 5:**
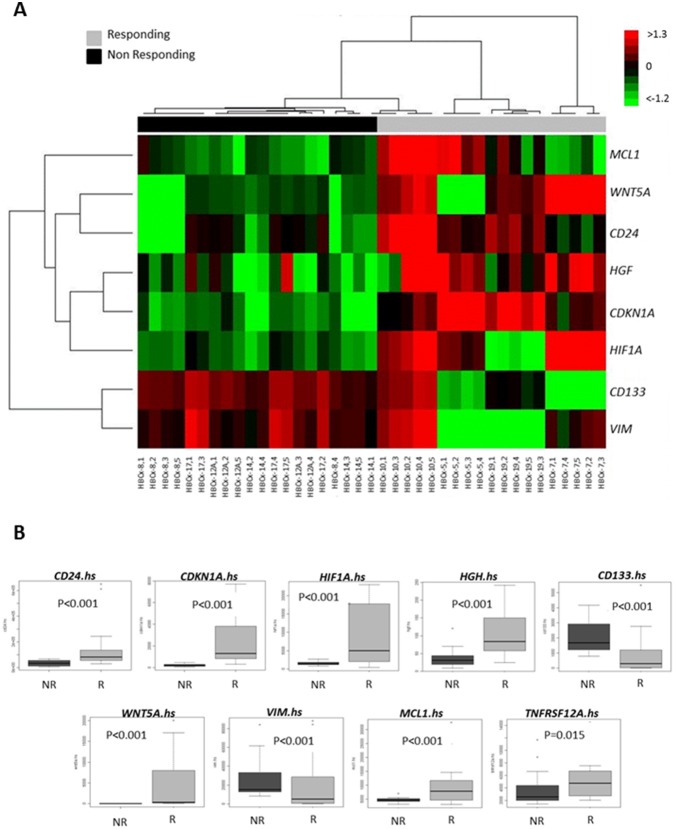
Predictive markers of response to PDL192. **A.** Genes that expression significantly influenced response to PDL192 and *TWEAKR/TNFRSF12A.*hs gene expression in responding and no responding models. (R: Responding; NR: non responding) **B.** Hierarchical clustering of the 8 best predictive genes.

Using a “hypothesis-driven” research approach, the expression levels of 57 candidate genes (49 human, 8 murine genes), known to be involved in various cellular and molecular mechanisms associated with TweakR, were measured by RT-QPCR in the control group of eight xenograft models [21]. Five tumors per model were analyzed. The “hypothesis-driven” RT-QPCR approach is an interesting alternative to complementary DNA (cDNA) microarrays for gene profiling. In particular, cDNA microarrays can be used to test thousands of genes at a time, while RT-PCR is a more accurate and quantitative assay method applicable to smaller number of selected genes [34]. Moreover, inclusion of both human and murine genes allowed the determination of the relative contribution(s) of tumor and/or stroma to the drug response signature what cannot be done with microarrays. Among the 8 models tested, 4 of them were considered as responding (HBCx-5, HBCx-7, HBCx-10, and HBCx-19) and the 4 others as non-responding to PDL192 (HBCx-8, HBCx-12A, HBCx-14, and HBCx-17). Fourteen human genes (*ALDH1A1*, *CXCL10*, *CXCL12*, *GLI1*, *IL1A*, *IL1B*, *IL6*, *MMP1*, *MMP9*, CD31/*PECAM1*, *SELE*, *SLUG*, *VEGFR2, CD44)* and one murine gene *(vegfr1)* were eliminated from the analysis because they were invariant in all studied models or expressed in only one model. To determine whether a gene or a group of genes were associated with response to PDL192 treatment, an ANOVA test was used on all included tumor samples. Twenty-three genes among the 42 remaining genes were associated with response to PDL192 treatment (Table S4). To improve the stability of the signature of predictive genes, we repeated the test 30 times on subsets created from the training data, and identified 8 genes systematically significantly associated to the response to treatment, i.e. *CD24*, *CDKN1A*, *HIF1A*, *MCL1*, *PROM1* (CD133), *VIM*, *HGF* and *WNT5A* (Fig. 5A). All of them were human genes, indicating that the signature of response was specifically a result of differential expression by the epithelial tumor cells. Hierarchical clustering containing the 8 identified genes defined two groups corresponding to responding and non-responding tumor xenografts (Fig. 5B). Six of the 8 genes were up-regulated in most of the responding models (*MCL1, WNT5A, CD24, HGF, CDKN1A* and *HIF1A*), the 2 remaining genes (*CD133* and *VIM*) were down-regulated in responding PDXs. Human *TNFRSF12A* expression was included in the 57 analyzed candidate genes but was not brought out by the signature (Fig. 5A). However, *TNFRSF12A* expression was significantly higher in responder than in non-responder models of PDX. This result confirms the role of TweakR expression in PDL192 response (Fig. 5B).

## Discussion

In this study, we have shown that (i) TWEAK receptor was expressed in about half of human breast cancers, (ii) the anti-TweakR antibody PDL192 was efficacious in 4 of 9 TweakR-positive patient-derived breast cancer xenografts, and (iii) a predictive eight gene signature of response was defined from responding and resistant PDXs that could be applied to a large cohort of breast cancer patients.

Our IHC study of TweakR expression in breast cancer patient tumor samples covered the four major sub-types of breast carcinomas, i.e. ERBB2 positive, basal-like, luminal A, and luminal B tumors, and concluded that TweakR expression was correlated with ER/ERBB2 coexpression but not with ER or ERBB2 alone. Although our result confirmed previously reported observations in term of global expression of the receptor, we did not find that TweakR expression was correlated to ERBB2 expression alone, as others had found [24], [32], perhaps due to the quite small number of patients analyzed in our population and variability between cohorts. In contrast, our prognostic study tended to show a linkage between poor overall survival of BC patients and positive TweakR expression, an observation that was consistent with previous results [32].

Nevertheless, one remaining issue is the determination of the threshold of TweakR expression that defines positive and negative tumors. This issue is one that must be taken into consideration for each new expression marker that impacts the decision of a targeted therapeutic treatment. Whereas in this study we defined the staining threshold at the median value of the TweakR staining score, in other studies, the threshold has been defined as 25% of stained epithelial cells [9], [24]. The criteria of threshold definition have not been formally established and should be adapted to each biomarker. Hence, we have observed that the efficacy of the anti-TweakR antibody PDL192 in TweakR-positive BC PDXs did not correlate with the TweakR level expression or its membranous localization. Indeed, the HBCx-10 PDX, that was defined by membranous expression and a H-score of 20, was highly sensitive to PDL192, as was the HBCx-19 PDX, which was characterized by cytoplasmic staining and a H-score of 190. These observations strongly showed that, besides TweakR expression and its cellular localization, some others factors are involved in the *in vivo* response to PDL192 antibody.

Identification of predictive markers of response is of high interest in the management of cancer patients: it would reduce patients’ receiving ineffective treatments and delay for other potential curative therapies, reduce unacceptable toxicities and would also reduce the cost of treatments. Hence, such an issue is included earlier and earlier in the strategy of new anticancer compound development. Nevertheless, predictive markers of response are often identified only after clinical trials and retrospective molecular reviews. Our approach aimed to define preclinical predictive markers in sensitive and resistant TweakR-positive PDXs that have been shown to replicate the molecular characteristics of human cancers [35], [36] and clinical responses [36]. In our study, specifically, we directed the identification of a predictive gene signature based on well-known mechanisms of the tested drug (“hypothesis-driven” research approach), which therefore decreased the risk of irrelevant biomarkers being selected. Using this approach, our study was performed on a selection of genes belonging to signaling pathways known to be involved in response to the PDL192 antibody, including the TweakR pathway, apoptosis, the NFκB pathway, proliferation, as well as molecular processes involved in the progression of breast cancers, including proliferation, migration/invasion, vascularization, and epithelial-mesenchymal transition (EMT) (Table S4).

Our study defined a predictive signature of eight genes: (i) *MCL1* and (ii) *CDKN1A* whose increased expression decreases apoptotic induction, (iii) *HIF1A* that has been shown to be correlated with *TWEAK* expression in experimental conditions of hypoxia [37], (iv) *WNT5A* which has been shown to be induced by TNF-α exposure [38], [39], and (v) *HGF*, the ligand of MET receptor. More interestingly, PDL192 response was found to be impacted by one EMT-related gene (vi) (*VIM*) and two cancer stem cell-related genes (vii and viii) (*CD24* and *CD133*), both processes of high importance in breast cancer progression [40], [41], [42]. Indeed, our data suggests that a low proportion of cancer stem cells and a low proportion of “mesenchymal” cells increased the likelihood of response to anti-TweakR antibody *in vivo*. Thus, it may be of interest to combine a TweakR targeting agent with an EMT-directed treatment to induce mesenchymal-epithelial transition in tumor cells and thus establish a more favorable condition for anti-TweakR therapy.

Another study has also investigated gene expression modifications under targeting of the Tweak-mediated signaling pathway [17]. After *in vitro* stimulation by the Tweak ligand, tumor cells were secondly treated by the antagonistic anti-TWEAK antibody RG7212. Hence, Yin et al. showed various modifications that reinforced our data, i.e. impact on genes encoding critical protein regulators of the NF-κB pathway (*TRAF1*, *BRIC3*, *NFκB2* and *NFκBIE*), apoptosis (*BRIC3, Bcl-X_L_*), or cytokine and chemokine involved in inflammation or metastasis processes. Finally, Tweak signaling was described to lead up-regulation of various anti-apoptotic genes such as *Bcl-X_L_* [43]. Based on the observation that common genes have been found in both *in vitro* Tweak-activated cells and *in vivo* responding PDXs, we can hypothesize that our responding models may have a constitutive activation of the Tweak-dependent pathway.

In conclusion, we have clearly demonstrated that Tweak targeting constitutes a promising therapeutic approach in breast cancer patients that could be directed by a relevant predictive gene signature of response. From this signature, it might now be of interest to determine new therapeutic associations that will be able to optimize TweakR targeting and increase response to treatment. The time has come to define personalized anticancer therapy using both tumor molecular features and predictive markers of therapeutic efficacy.

## Supporting Information

Figure S1 **TweakR staining distributions in the overall patient’s tumors (E, F) and in patient’s tumors according to their breast cancer sub-groups (D, E, F) and xenografts (G, H, I) according to the intensity (A, C, E) and the proportion of positive tumor cells (B, D, F).**
(TIF)

Table S1 **Primer Sequences.**
(PDF)

Table S2 **TweakR expression in studied breast cancer PDXs.**
(PDF)

Table S3 **Correlation between TweakR H-score and in vivo responses to PDL192.**
(PDF)

Table S4 **Predictive genes of response to PDL192.**
(PDF)
