# Supplementary figures and images for: Predictive Gene Signature of Response to the Anti-TweakR mAb PDL192 in Patient-Derived Breast Cancer Xenografts
Source: PLoS One. 2014 Nov 6;9(11):e104227. doi: 10.1371/journal.pone.0104227 (PMC4222831; doi:10.1371/journal.pone.0104227)

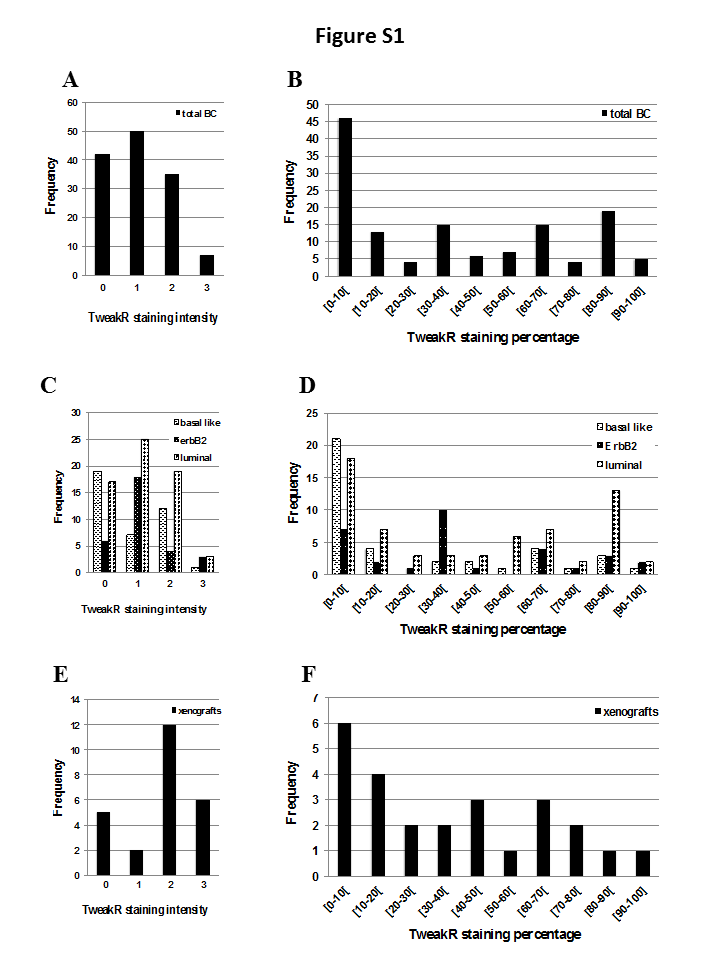

Supplement: Figure S1 — TweakR staining distributions in the overall patient’s tumors (E, F) and in patient’s tumors according to their breast cancer sub-groups (D, E, F) and xenografts (G, H, I) according to the intensity (A, C, E) and the proportion of positive tumor cells (B, D, F). (TIF) [file pone.0104227.s001.tif]
