# Supplementary material for: Predictive Gene Signature of Response to the Anti-TweakR mAb PDL192 in Patient-Derived Breast Cancer Xenografts
Source: PLoS One. 2014 Nov 6;9(11):e104227. doi: 10.1371/journal.pone.0104227 (PMC4222831; doi:10.1371/journal.pone.0104227)
Supplement: Table S1 — Primer Sequences. (PDF) [file pone.0104227.s002.pdf]

Table S1: Primer Sequences

Genes	Species	Upper primer (5' to 3')	Lower primer (5' to 3')
ALDH1A1	HS	CGTCTGCTGCTGGCGACAAT	ACCTGCACAGTAGCGCAATGTTT
AURKA	HS	GCATTTCAGGACCTGTTAAGGCTA	TGCTGAGTCACGAGAACACGTTT
BCL2	HS	CCCCTGGTGGACAACATCGC	AGTTCCACAAAGGCATCCCAG
BCLX-L	HS*	TGAATGACCACCTAGAGCCTTGGA	GGAACCAGCGGTTGAAGCGT
CD133	HS	TGGTCCAACAGGGCTATCAATC	TTCAAGACCCTTTTGATACCTGCTA
CD24	HS	ACTGCTCCTACCCACGCAGATT	GGGAGGAGTTACTTGAAGTTCCAGTT
CD31/PECAM1	HS	CTGCTGACCCTTCTGCTCTGTTC	GGCAGGCTCTTCATGTCAACACT
cd31/pecam1	MM	GACTCACGCTGGTGCTCTATGC	TCAGTTGCTGCCCATTCATCA
CD44	HS	CTTTCAATAGCACCTTGCCCAC	CCCTTCTATGAACCCATACCTGC
CDH1	HS	CGCATTGCCACATACACTCTCTT	TCGGGCTTGTTGTCATTCTGAT
CXCL1/GRO1	HS	AACCCCAAGTTAGTTCAATCTGGA	CATGTTGCAGGCTCCTCAGAA
CXCL10/IP10	HS	CTGACTCTAAGTGGCATTCAAGGAG	GGTTGATTACTAATGCTGATGCAGG
CXCL12	HS	CTGTGCCCTTCAGATTGTAGCC	CAGGTACTCCTGAATCCACTTTAGC
cxcl12	MM	CGCTCTGCATCAGTGACGGTAA	GACGTTGGCTCTGGCGATGT
CXCR4	HS	CTTCATCTTTGCCAACGTCAGTG	AACCATGATGTGCTGAAACTGGA
cxcr4	MM	GTCAGCCAGGGGGACATCAGT	GAATTGAAACACCACCATCCACAG
FASL	HS	AGTGGCCCATTTAACAGGCAAGT	CTCCAGAAAGCAGGACAATTCCAT
FGF2	HS	AGAAGAGCGACCCTCACATCAA	GGTAACGGTTAGCACACACTCCTT
GLI1	HS	CCAACTCCACAGGCATACAGGAT	CACAGATTCAGGCTCACGCTTC
GLUT1	HS	CCGGGCCAAGAGTGTGCTAA	CCGCATCATCTGCCGACTCT
HGF	HS*	CCCTGGTGTTTCACAAGCAATC	GGTCATGCATTCAACTTCTGAACA
HIF1A	HS	GATTTTGGCAGCAACGACACA	GGTGAGGGGAGCATTACATCATTA
ICAM	HS	GCGGCTGACGTGTGCAGTAAT	GGCGCCGGAAAGCTGTAGA
IL1A	HS	CCTGAATGACGCCCTCAATCAA	CCCATGTCAAATTTCACTGCTTCA
IL1B	HS	GCAAAAAAGCTTGGTGATGTCTG	AAGGACATGGAGAACACCACTTGT
IL6	HS	CAATCTGGATTCAATGAGGAGAC	CTCTGGCTTGTTCCTCACTACTC
IL8	HS	CACCGGAAGGAACCATCTCACTGT	TCCTTGGCAAAACTGCACCTTCA
MCL1	HS*	AACAAAGAGGCTGGGATGGG	TACTCCAGCAACACCTGCAAAA
MET	HS	ATGGGTCAATTCAGCGAAGTCC	GATCGAGAAACCACAACCTGCAT
MKI67	HS	ATTGAACCTGCGGAAGAGCTGA	GGAGCGCAGGGATATTCCCTTA
MMP1	HS	GGCTTGAAGCTGCTTACGAATTT	ACAGCCCAGTACTTATTCCCTTTGA
MMP11	HS	GCACCCGGCGTGTAGACAGT	GCAGGAAGTAGGCATAGCCATCA
MMP9	HS	GCATCCGGCACCTCTATGGTCCT	GAGCCGTGGGCTGCGGTGT
mmp9	MM	CTGCATTTCTTCAAGGACGGTTGGTA	GCCACGTGCGGGCAGTAAGG
NEK2	HS	CCCTGTATTGAGTGAGCTGAAACTG	GCTCCTGTTCTTTCTGCTCCAAT
p21/CDKN1A	HS	CAGGTGGACCTGGAGACTCTCA	GCGTTTGGAGTGGTAGAAATCTGT
PLAUR	HS	ACACCTGCGTCCCAGCCTCT	CGCACTCTTCCACACGGCA
PLK1	HS	GCAGATCAACTTCTTCCAGGATCA	CGCTTCTCGTCGATGTAGGTCA
SELE	HS	CACATCTCAGGGACAATGGACAGA	GCTTGAACATTTTACCACTTGGCA
SLUG	HS	GCGAACTGGACACACATACAGTGA	GCAGCGGTAGTCCACACAGTGA
SNAIL	HS	GCTGCAGGACTCTAATCCAGAGTT	GACAGAGTCCCAGATGAGCATTG
TBP	HS	AGAACAACAGCCTGCCACCTTAC	GGGAGTCATGGCACCCTGAG
Tbp	MM	CCCTTGTACCCTTCACCAATGAC	TCACGGTAGATACAATATTTTGAAGCTG
TBP total	HS + MM	TGCACAGGAGCCAAGAGTGAA	CACATCACAGCTCCCCACCA
TCF4	HS	TCACCGGCACACATTGTCTCTAA	GGCGTGAAGTGTTCATTGCTGTA
TGFB1	HS	GTCACCCGCGTGCTAATGGT	TTCTCGGAGCTCTGATGTGTTGA

TNF	HS	GCCCAGGCAGTCAGATCATCTT	CCTCAGCTTGAGGGTTTGCTACA
TNFAIP3/A20	HS	CATCTCATCAATGCCGCAAAGTT	TCCTGCCATTTCTTGTACTCATGCT
TNFRSF12A	HS	TGGGGCTGCTTTCTGGCTT	GTCTCCTCTATGGGGGTGGTGA
Tnfrsf12A	MM	GGTTTTGGCGCTGGTTTCTAGTT	CACCAGTCTCCTCTATGGGGGTAGTA
TRAF2	HS	CAGTTCGGCCTTCCCAGATAA	CCTTCGTGGCAGCTCTCGT
TRAF6	HS*	CTCATCAGAGAACAGATGCCTAATCA	CTTGTAGGTGGCGTGCCAAGT
TWIST	HS	CGCCCCGCTCTTCTCCTCT	TGGACACGTCCTGCATCATCTCT
VEGFA	HS	CAGAAGGAGGAGGGCAGAATCAT	TGGCAGTAGCTGCGCTGATAGA
Vegfa	MM	GCACTGGACCCTGGCTTTACT	ATGAACTTGATCACTTCATGGGACT
VEGFR1	HS	ATCATTCCGAAGCAAGGTGTGAC	TCCTTCTATTATTGCCATGCGCT
Vegfr1	MM	CCACAATCACTCCAAAGAAAGGTATG	TCAATTCTGTTTCCTAAGTTGCTGCT
VEGFR2/KDR	HS	TCAACGTGTCACTTTGTGCAAGATAC	CCAGGAAATTCTGTTACCATCAGGA
Vegfr2/Kdr	MM	TCAATGTGTCTCTTTGCGCTAGGTAT	GGGAGAGTAAAGCCTATCTCGCTGT
VIM	HS*	CTCCCTCTGGTTGATACCCACTC	AGAAGTTTCGTTGATAACCTGTCCA
WNT5A	HS	AGCCAATTCTTGGTGGTCGCTA	TGCAGAGAGGCTGTGCTCCTATAA

Abbreviations: HS, *Homo Sapiens*; MM, *Mus Musculus*.

* Five primer couples were not completely specific to the human sequences. Only 7% of murin (mean) (Supplemental Table S1) cells were found in the xenograft and this recognition was lower than that of human mRNA, the amplification products of these couples can then be considered as mainly representative of human expression.
