# Supplementary material for: Predictive Gene Signature of Response to the Anti-TweakR mAb PDL192 in Patient-Derived Breast Cancer Xenografts
Source: PLoS One. 2014 Nov 6;9(11):e104227. doi: 10.1371/journal.pone.0104227 (PMC4222831; doi:10.1371/journal.pone.0104227)
Supplement: Table S2 — TweakR expression in studied breast cancer PDXs. (PDF) [file pone.0104227.s003.pdf]

Table S2: TweakR expression in studied breast cancer PDXs

Tumor graft	EPITHELIAL CELLS				STROMA		Tumor Type
	staining	%	intensity	H score	staining	intensity	
HBCx-1	no	0	0	0	vessels	2	Basal
HBCx-2	membrane	85	3	255	fibroblats	2	Basal
HBCx-3	cytoplasm	70	2	140	no	0	Luminal
HBCx-5	membrane	50	2	100	no	0	ErbB2
HBCx-7	membrane	60	3	180	vessels	3	Basal
HBCx-8	membrane	60	3	180	vessels	3	Basal
HBCx-10	membrane	10	2	20	vessels	3	Basal
HBCx-11	no	0	0	0	vessels	2	Basal
HBCx-13A	cytoplasm	40	3	120	vessels	3	ErbB2
HBCx-13B	cytoplasm	20	2	40	0	0	ErbB2
HBCx-14	membrane	30	2	60	vessels	3	Basal
HBCx-12A	membrane	45	3	135	vessels	3	Basal
HBCx-12B	membrane	10	2	20	vessels	3	Basal
HBCx-19	cytoplasm	95	1.5	142.5	0	0	Basal
HBCx-17	membrane	15	2	30	vessels	3	Basal
HBCx-21	no	0	0	0	0	0	Luminal
HBCx-22	cytoplasm	60	2	120	vessels	3	Luminal
HBCx-23	cytoplasm	20	2	40	vessels	2	Basal
HBCx-24	no	0	0	0	vessels	2	Basal
HBCx-26	cytoplasm	40	2	80	0	0	Basal
HBCx-28	membrane	15	3	45	vessels	2	Basal
HBCx-29	membrane	35	2	70	vessels	3	Luminal
HBCx-31	no	0	0	0	vessels	2	Basal
HBCx-33	cytoplasm	5	1	5	vessels	1	Basal
HBCx-36	cytoplasm	70	2.5	175	vessels	3	Luminal
Tweak-R positive tumor grafts : 16/25 (64%)							
