# Supplementary material for: Predictive Gene Signature of Response to the Anti-TweakR mAb PDL192 in Patient-Derived Breast Cancer Xenografts
Source: PLoS One. 2014 Nov 6;9(11):e104227. doi: 10.1371/journal.pone.0104227 (PMC4222831; doi:10.1371/journal.pone.0104227)
Supplement: Table S3 — Correlation between TweakR H-score and in vivo responses to PDL192. (PDF) [file pone.0104227.s004.pdf]

Table S3: Correlation between TweakR H-score and *in vivo* responses to PDL192

PDXs	Histological Type	p53 status	TweakR H-score	PDL192 inhibitory effect
HBCx-5	ErbB2	*WT*	100	**Yes**
HBCx-7	basal	*WT*	180	**Yes**
HBCx-8	basal	*WT*	180	No
HBCx-10	basal	*M*	20	**Yes**
HBCx-12A	basal	*M*	135	No
HBCx-12B	basal	*M*	20	No
HBCx-14	basal	*M*	60	No
HBCx-17	basal	*M*	30	No
HBCx-19	basal	*M*	190	**Yes**

Abbrevations: WT, wild-type; M, mutated.
