# Supplementary material for: Predictive Gene Signature of Response to the Anti-TweakR mAb PDL192 in Patient-Derived Breast Cancer Xenografts
Source: PLoS One. 2014 Nov 6;9(11):e104227. doi: 10.1371/journal.pone.0104227 (PMC4222831; doi:10.1371/journal.pone.0104227)
Supplement: Table S4 — Predictive genes of response to PDL192. (PDF) [file pone.0104227.s005.pdf]

Table 4S: Predictive genes of response to PDL192

	mRNA	R/NR models	n	mean	sd	corrected p
human **PDL192-targeted mRNA**	*TWEAKR/TNFRSF12A*	NR<br>R	20<br>19	3600<br>5480	2766<br>3464	**0.0105**
murin **PDL192-targeted mRNA**	*TWEAKR/TNFRSF12A*	NR<br>R	20<br>19	1389<br>1253	515<br>546	0.3262
**Metabolism** human mRNA	*GLUT1*	NR<br>R	20<br>19	3638<br>9335	1928<br>11926	**0.0096**
**NFKB** human mRNA	*CXCL10/IP10*	*Invariant or not exprimed*				
	ICAM	NR<br>R	20<br>19	159<br>325	151<br>258	0.1318
	SELE	*Invariant or not exprimed*				
	CXCL1	NR<br>R	20<br>19	996<br>4583	1008<br>8156	**0.0016**
	TNFAIP3/A20	NR<br>R	20<br>19	707<br>948	433<br>968	0.9157
**Proliferation** human mRNA	*MKI67*	NR<br>R	20<br>19	4832<br>8521	2970<br>10298	0.208
	PLK1	NR<br>R	20<br>19	1857<br>1697	1198<br>1383	0.608
	AURKA	NR<br>R	20<br>19	2303<br>4072	1392<br>2610	**0.0229**
	NEK2	NR<br>R	20<br>19	1403<br>1774	717<br>1924	1.000
	p21/CDKN1A	NR<br>R	20<br>19	219<br>2580	112<br>2474	**$< 10^{-7}$**
**Apoptosis** human mRNA	*BCL-XL*	NR<br>R	20<br>19	932<br>3969	468<br>4669	**0.00002**
	BCL2	NR<br>R	20<br>19	167<br>194	133<br>124	0.224
	p21/CDKN1A	NR<br>R	20<br>19	219<br>2580	112<br>2474	**< 10-7**
	TRAF2	NR<br>R	20<br>19	870<br>1078	306<br>712	0.334
	TRAF6	NR<br>R	20<br>19	136<br>220	92<br>133	**0.03**
	MCL1	NR<br>R	20<br>19	4679<br>9755	884<br>7603	**0.000001**
**Migration/Invasion** murin mRNA	*MMP9*	NR<br>R	20<br>19	3904<br>2997	4368<br>4448	**0.003**
	CXCL12	NR<br>R	20<br>19	1210<br>1297	377<br>853	0.558
	CXCR4	NR<br>R	20<br>19	5149<br>6297	3238<br>3483	0.251

Table 4S: Predictive genes of response to PDL192

	mRNA	R/NR models	n	mean	sd	corrected p
**Migration/Invasion** human mRNA	*MMP9*	*Invariant or not exprimed*				
	MMP1	*Invariant or not exprimed*				
	MMP11	NR	20	38	31	**0.001**
		R	19	87	63	
	CXCL12	*Invariant or not exprimed*				
	CXCR4	NR	20	942	1097	**0.004**
		R	19	3525	4040	
	PLAUR	NR	20	1389	1180	**0.013**
		R	19	510	586	
	CD44	*Invariant or not exprimed*				
	CD24	NR	20	35645	22534	**0.000005**
		R	19	150889	2E+05	
**EMT** human mRNA	***VIM***	NR	20	25495	21703	**< 10-7**
		R	19	21005	28983	
	CDH1	NR	20	9982	4048	**0.0000**
		R	19	17617	20166	
	TWIST1	NR	20	114	163	0.6827
		R	19	69	79	
	SNAIL	NR	20	298	183	**0.0005**
		R	19	227	246	
	SLUG	*Invariant or not exprimed*				
**Vascularization** human mRNA	*VEGFA*	NR	20	3992	2459	**0.055**
		R	19	11592	15105	
	VEGFR1	NR	20	2564	1192	0.1313
		R	19	3353	1795	
	VEGFR2	*Invariant or not exprimed*				
	CD31 /PECAM1	*Invariant or not exprimed*				
	HIF1A	NR	20	1513	497	**< 10-7**
		R	19	9002	8208	
	FGF2	NR	20	103	82	**0.0009**
		R	19	423	575	
**Vascularization** murin mRNA	*VEGFA*	NR	20	3072	1055	0.055
		R	19	2422	1181	
	VEGFR1	*Invariant or not exprimed*				
	VEGFR2	NR	20	3063	1576	0.516
		R	19	3394	1575	
	CD31 /PECAM1	NR	20	8601	4025	0.951
		R	19	8759	4158	
**Immunity** human mRNA	*TNF*	NR	20	70	87	0.2243
		R	19	42	56	
	IL8	NR	20	1269	2154	0.31
		R	19	29038	52745	
	IL1A	*Invariant or not exprimed*				
	IL1B	*Invariant or not exprimed*				
	IL6	*Invariant or not exprimed*				

Table 4S: Predictive genes of response to PDL192

	mRNA	R/NR models	n	mean	sd	corrected p
**Stem cell markers** human mRNA	***CD133/PROM1***	NR<br>R	20<br>19	2034<br>1057	1094<br>1678	**< 10-7**
	CD44	NR<br>R	20<br>19	6898<br>9385	5282<br>6453	0.5164
	CD24	NR<br>R	20<br>19	35645<br>150889	22534<br>2E+05	**0.000005**
	ALDH1A1	*Invariant or not exprimed*				
	TCF4					
	GLI1					
**Signaling Pathways** human mRNA	*MET*	NR<br>R	20<br>19	2185<br>2137	3022<br>3491	**0.0105**
	HGF	NR<br>R	20<br>19	36<br>102	26<br>60	**0.00003**
	TGFB1	NR<br>R	20<br>19	360<br>411	474<br>515	0.560
	WNT5A	NR<br>R	20<br>19	11<br>4550	7<br>7224	**< 10-7**
	TCF4	NR<br>R	20<br>19	1231<br>787	1056<br>738	0.202
	GLI1	*Invariant or not exprimed*				
